# Community Knowledge, Attitudes, and Practices Regarding Ebola Virus Disease — Five Counties, Liberia, September–October, 2014

**Published:** 2015-07-10

**Authors:** Miwako Kobayashi, Karlyn D. Beer, Adam Bjork, Kevin Chatham-Stephens, Cara C. Cherry, Sampson Arzoaquoi, Wilmot Frank, Odell Kumeh, Joseph Sieka, Adolphus Yeiah, Julia E. Painter, Jonathan S. Yoder, Brendan Flannery, Frank Mahoney, Tolbert G. Nyenswah

**Affiliations:** 1 Epidemic Intelligence Service, CDC; 2 National Center for Immunization and Respiratory Diseases, CDC; 3 National Center for Emerging and Zoonotic Infectious Diseases, CDC; 4 Division of Global HIV/AIDS, Center for Global Health; 5 National Center for Environmental Health, CDC; 6 National Park Service, Biological Resources Division, Wildlife Health Branch/Office of Public Health, Fort Collins, Colorado; 7 Bong County Health Office; 8 Sinoe County Health Office; 9 Maryland County Health Office; 10 River Gee County Health Office; 11 Margibi County Health Office; 12 Global Immunization Division, Center for Global Health, CDC; 13 Ministry of Health, Liberia

As of July 1, 2015, Guinea, Liberia, and Sierra Leone have reported a total of 27,443 confirmed, probable, and suspected Ebola virus disease (Ebola) cases and 11,220 deaths (1). Guinea and Sierra Leone have yet to interrupt transmission of Ebola virus. In May, 2015, Liberia successfully achieved Ebola transmission-free status (2), with no new Ebola cases occurring during a 42-day period; however, new Ebola cases were reported beginning June 29, 2015 (1). Local cultural practices and beliefs have posed challenges to disease control (3), and therefore, targeted, timely health messages are needed to address practices and misperceptions that might hinder efforts to stop the spread of Ebola. As early as September 2014, Ebola spread to most counties in Liberia. To assess Ebola-related knowledge, attitudes, and practices (KAP) in the community, CDC epidemiologists who were deployed to the counties (field team), carried out a survey conducted by local trained interviewers. The survey was conducted in September and October 2014 in five counties in Liberia with varying cumulative incidence of Ebola cases. Survey results indicated several findings. First, basic awareness of Ebola was high across all surveyed populations (median correct responses = 16 of 17 questions on knowledge of Ebola transmission; range = 2–17). Second, knowledge and understanding of Ebola symptoms were incomplete (e.g., 61% of respondents said they would know if they had Ebola symptoms). Finally, certain fears about the disease were present: >90% of respondents indicated a fear of Ebola patients, >40% a fear of cured patients, and >50% a fear of treatment units (expressions of this last fear were greater in counties with lower Ebola incidence). This survey, which was conducted at a time when case counts were rapidly increasing in Liberia, indicated limited knowledge of Ebola symptoms and widespread fear of Ebola treatment units despite awareness of communication messages. Continued efforts are needed to address cultural practices and beliefs to interrupt Ebola transmission.

From September 17 through October 11, 2014, a field team of CDC epidemiologists, in partnership with local trained volunteers, conducted an Ebola KAP survey in five counties in Liberia (Bong, Margibi, Maryland, River Gee, and Sinoe) with varying cumulative incidence of Ebola cases per 100,000 population (Figure). Two of the five counties, Bong and Margibi, had high cumulative incidence and three southeastern counties, Maryland, River Gee, and Sinoe, had low cumulative incidence. Bong and Margibi reported Ebola cases early in the outbreak (March 2014), and as of September 17 had reported 482 (Bong) and 229 (Margibi) total (confirmed, probable, and suspected) cumulative cases (4). Both counties already had implemented Ebola-response activities, such as case investigation, contact tracing, and safe burial procedures. An Ebola treatment unit opened in Bong shortly before the survey was conducted. In contrast, Maryland, River Gee, and Sinoe counties, which reported their first Ebola cases in August 2014, had reported cumulative counts of eight, 12, and six Ebola cases, respectively (4). These three counties had limited experience with outbreak response (5). The KAP survey took place as response and preparedness efforts were scaling up rapidly in the southeast (5). Survey areas were selected from three community categories: 1) population centers, such as county capitals and commercial districts; 2) communities with high Ebola incident cases within the county, such as communities reporting the highest number of persons in whom Ebola was newly diagnosed, or those in southeastern counties where cases were being reported; or 3) communities that were at risk for receiving Ebola cases, such as those located along main roads.

Across all five counties, more than 50 survey areas were selected. The goal was to survey 60–100 persons per county. Survey respondents were members of the general public from the communities, including Ebola survivors. The field team trained literate volunteers among local students or county health office staff members to be surveyors. The volunteer surveyor group then recruited participants and administered survey questionnaires using a standardized form across all sites. Subject matter experts reviewed the survey’s content before it was administered and local surveyors pilot-tested the surveys. Surveyors were instructed to share accurate information about Ebola after the survey. Surveyors approached community members in public areas, including residential and commercial zones, to solicit participation in the KAP survey. From a central point in populated areas in each district, surveyors randomly chose a direction to approach community members, resulting in a non-probability sample of persons encountered in each county. Surveys were identical for all survey areas, and administered orally, in English. Whenever possible, individual surveys were conducted away from other persons.

Interviewers collected demographic data on sex, age, highest level of education, and occupation. Respondents were asked whether they agreed or disagreed with a total of 38 statements. The statements were divided into scored and non-scored sections. The scored section contained statements (N = 33) with responses scored using the contents of local health messages as a reference. The other statements (N = 5) were placed in the non-scored section. The scored section was further divided into three KAP categories: 1) Ebola knowledge (17 questions designed to gauge respondents’ understanding of Ebola transmission), 2) Ebola attitudes (nine questions on perceptions about Ebola, Ebola patients, and treatment centers), and 3) Ebola practices (seven questions used to assess respondents’ anticipated practices if they or an acquaintance were to become symptomatic). Statements were scored “correct” if they were consistent with Liberia’s Ministry of Health (MOH) health messaging at the time of the survey. The five non-scored statements were designed to assess respondents’ subjective fears regarding Ebola, Ebola patients, or treatment centers. All responses were handwritten by surveyors in printed forms, and collected information was entered electronically into Excel by the CDC field team.

Responses were summarized by county groups, based on Ebola incidence (high [Margibi and Bong] versus low [Maryland, River Gee, and Sinoe]). Univariate analyses were performed to assess differences in knowledge, attitudes, and practices among participants. Wilcoxon rank-sum tests were used for ordinal data and Chi-square tests or Fisher’s exact tests were used for categorical data.

Overall, there were 609 respondents from the five counties (Bong [n = 212], Margibi [n = 126], Maryland [n = 106], River Gee [n = 97], and Sinoe [n = 68]).* Although no official records were kept, the average response rate was estimated at >90%, based on the survey teams’ experience with the refusal rate of persons approached. Among all respondents, 291 (48.2%) were women, and the median age was 32 years (range = 12–99). A majority (58.4%) of respondents had completed middle school education or higher. Of the 33 scored statements, overall, respondents answered correctly a median of 16 (range = 2–17) of 17 Ebola knowledge questions. The correct responses for attitudes (median = 7 of 9 questions correct; range = 1–9) and anticipated practices (median = 7 of 7 questions correct; range = 1–7) also were high, and did not differ by county (Table 1).

The knowledge areas where low-incidence counties scored lower were related to the questions on Ebola transmission, such as eating bush meat and attending burials (where persons might come in contact with the body) of Ebola patients (Table 2). In addition, more respondents from low-incidence counties believed that a curse or spell could result in Ebola transmission, compared with those from high-incidence counties (34.8% versus 7.4%; p<0.01). Among respondents from all five counties, >30% agreed that a person can get Ebola from a healthy (asymptomatic) person. Scores were lower in a few key areas: respondents across all counties were not confident in their ability to identify Ebola symptoms, were fearful of survivors, and were afraid that if they went to an Ebola treatment unit, they would not be allowed to see their family. One statistically significant difference in attitude between high- and low-incidence counties was a fear of cured patients (34.6% [high-incidence] and 47.8% [low-incidence], p<0.01) and a fear that a person would not be allowed to see their family if they were admitted to an Ebola treatment unit (37.9% [high-incidence] and 61.6% [low-incidence], p<0.01) (Table 2).

Responses to the five non-scored statements on Ebola-related fears showed that a large proportion (>90%) of respondents feared Ebola patients and persons who live with Ebola patients (Table 3). Respondents in high-incidence counties were more fearful of these groups than those in low-incidence counties. Similarly, fear of Ebola treatment units was reported by more than half of respondents in both low- and high-incidence county groups; however, a significantly larger proportion from the low-incidence group reported fear of seeking care, and thought they would die if they sought care.

## Discussion

Overall, Ebola awareness was high, based on the median correct responses for the 33 statements in the scored section of the KAP survey. At the same time, this survey revealed several important areas of concern as Liberia sought to contain the Ebola epidemic. Across all counties, respondents were somewhat less able to correctly recognize Ebola symptoms or the transmission risk from asymptomatic persons; fear of Ebola patients and Ebola treatment units was prevalent. The fear of cured patients might partially be explained by the fact that community acceptance of survivors was not part of the initial set of Ebola health messages in Liberia. Targeted educational messages about Ebola virus transmission modes, how to protect oneself against Ebola, and the purpose of Ebola treatment units might help to alleviate some of these fears. In addition, recurrence of Ebola transmission in Liberia reinforces the need for ongoing vigilance and early detection of symptomatic persons.

The findings in this report are subject to at least two main limitations. First, the selection of communities within the counties was non-random. However, counties were selected in consultation with MOH, on the basis of priority for intervention at the time. As a result, the survey covered areas with varying levels of Ebola incidence. Second, a standardized form was used for the survey, but none of the responses was open-ended. Therefore, limited information was available beyond the binary agree or disagree responses.

The Ebola KAP is believed to be the first survey that was conducted during this Ebola outbreak to assess the effectiveness of initial Ebola messaging at the community level across a wide geographical area in Liberia. The recent recurrence of Ebola cases in Liberia highlights the continued risk for transmission in the region. Future health awareness activities, especially in Guinea and Sierra Leone where the epidemic is not fully contained, might benefit from emphasizing the signs and symptoms of Ebola, addressing fears about seeking treatment and placing additional focus on counties and communities where incidence of Ebola is low as a preparedness measure. A follow-up survey might be needed to assess the current Ebola awareness among the public more than a year after this Ebola outbreak began. Continued efforts are needed to address cultural practices and beliefs to interrupt Ebola transmission.

**Summary**

What is already known on this topic?

Local cultural practices and beliefs related to Ebola have presented challenges to controlling the current outbreak in West Africa. Community engagement is an important component of Ebola control.

What is added by this report?

Early in the epidemic, Ebola awareness was widespread within communities in Liberia, based on a knowledge, attitudes, and practices (KAP) survey. However, differences were observed between counties based on Ebola incidence. Areas of concerns include large numbers of participants not being confident with Ebola symptom identification, and existing fears of Ebola survivors and of Ebola treatment units.

What are the implications for public health practice?

Survey findings could be used to inform ongoing health awareness and messaging to address specific fears, misperceptions, and practices regarding Ebola. This study might offer useful insight for countries during Ebola containment efforts.

## Figures and Tables

**FIGURE:**
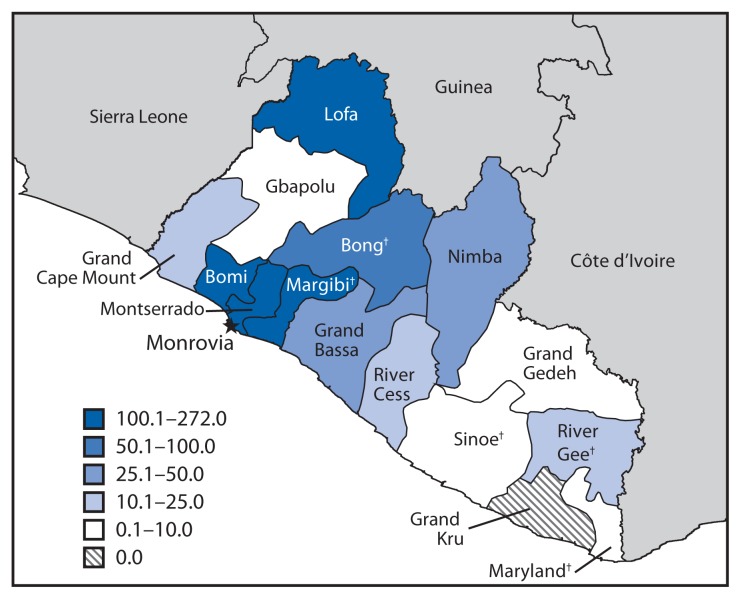
Cumulative incidence^*^ of Ebola virus disease, by county — Liberia, as of September 20, 2014 **Sources:** Liberia Ministry of Health. ^*^ Number of reported cases per 100,000 population. ^†^ Counties surveyed were Bong, Margibi, Maryland, River Gee, and Sinoe.

**TABLE 1:** Summary results of the scored section, Knowledge, Attitudes and Practices survey — Liberia, September 17–October 11, 2014

Categories/No.	Median score						p-value

	All respondents		High-incidence counties*		Low-incidence counties†		
		
	Score	(Range)	Score	(Range)	Score	(Range)	
Knowledge about Ebola transmission/17	16	(2–17)	16	(4–17)	15	(2–17)	<0.01
Attitudes/9	7	(1–9)	8	(3–9)	7	(1–9)	0.05
Anticipated practices/7	7	(1–7)	7	(3–7)	7	(1–7)	0.07

* High-incidence counties = Bong and Margibi.

† Low-incidence counties = Maryland, River Gee, and Sinoe.

**TABLE 2:** Questions, preferred responses, and participants’ responses, for the scored section of the Knowledge, Attitudes, and Practices survey — Liberia, September 17–October 11, 2014

Questions	MOHSW preferred response	Agreed among all respondents (N = 609)		Agreed in high-incidence counties* (n = 338)		Agreed in low-incidence counties† (n = 271)		p-value
		
		No.	(%)	No.	(%)	No.	(%)	
**Knowledge about transmission**								
I can get Ebola from a healthy (asymptomatic) person	Disagree	243	(40.0)	123	(36.5)	120	(44.3)	0.05
I can get Ebola from kissing a symptomatic person	Agree	593	(97.5)	328	(97.3)	265	(97.8)	0.72
I can get Ebola from sharing a spoon/fork with a symptomatic person	Agree	589	(96.7)	328	(97.0)	261	(96.3)	0.61
I can get Ebola from sleeping in the same bed as a symptomatic person	Agree	585	(96.5)	334	(98.8)	251	(93.7)	<0.01
I can get Ebola from cleaning up vomit from a symptomatic person	Agree	591	(97.2)	331	(98.2)	260	(95.9)	0.09
I can get Ebola from having sex with a symptomatic person, even if I wear a condom	Agree	591	(97.0)	333	(98.5)	258	(95.2)	0.02
I can get Ebola from cleaning up pee or poop from a symptomatic person	Agree	596	(97.9)	334	(98.8)	262	(96.7)	0.07
I can get Ebola from touching a dead person	Agree	569	(93.4)	321	(95.0)	248	(91.5)	0.09
I can get Ebola from washing a dead person	Agree	573	(94.1)	325	(96.2)	248	(91.5)	0.02
I can get Ebola from cleaning the sheets from a funeral of an Ebola patient	Agree	590	(96.9)	332	(98.2)	258	(95.2)	0.03
I can get Ebola from eating bush meat	Agree	492	(80.8)	290	(85.8)	202	(74.5)	<0.01
I can get Ebola from attending a burial of an Ebola patient	Agree	519	(85.5)	305	(90.5)	214	(79.3)	<0.01
A baby can get Ebola from breastfeeding from a symptomatic mother	Agree	591	(97.0)	336	(99.4)	255	(94.1)	<0.01
Fever is a symptom of Ebola	Agree	494	(81.5)	286	(84.9)	208	(77.3)	0.02
Hand washing can prevent transmission of Ebola	Agree	579	(95.2)	328	(97.3)	251	(92.6)	<0.01
Anyone can get Ebola (even healthy people)	Agree	574	(94.6)	328	(97.0)	246	(91.5)	<0.01
I can get Ebola if someone puts a curse/spell on me	Disagree	118	(19.5)	25	(7.4)	93	(34.8)	<0.01

Questions	MOH preferred response	Agreed among all respondents (N = 609)		Agreed in high-incidence counties* (n = 338)		Agreed in low-incidence counties† (n = 271)		p-value
		
		No.	(%)	No.	(%)	No.	(%)	
**Attitudes**								
Ebola is a real disease	Agree	596	(97.9)	333	(98.5)	263	(97.1)	0.21
Ebola is a serious disease	Agree	596	(98.0)	335	(99.1)	261	(96.7)	0.03
I am worried about getting Ebola	Agree	536	(88.0)	302	(89.4)	234	(86.4)	0.26
I am at risk for getting Ebola	Agree	524	(86.5)	298	(88.7)	226	(83.7)	0.07
I am afraid of people who have been cured of Ebola	Disagree	246	(40.5)	117	(34.6)	129	(47.8)	<0.01
I would know if I had Ebola symptoms	Agree	367	(61.0)	203	(60.8)	164	(61.2)	0.92
I know how to protect myself from getting Ebola	Agree	543	(89.5)	296	(87.6)	247	(91.8)	0.09
If I go to a treatment center, I will not be allowed to see my family	Disagree	293	(48.4)	128	(37.9)	165	(61.6)	<0.01
White people brought Ebola here	Disagree	73	(12.1)	37	(11.0)	36	(13.4)	0.37
**Anticipated Practices**								
If I got Ebola symptoms, I would seek treatment	Agree	587	(96.6)	337	(99.7)	250	(92.6)	<0.01
If I got Ebola symptoms, I know where to go for treatment	Agree	492	(80.9)	284	(84.0)	208	(77.0)	0.03
If I got Ebola symptoms, I would go to a traditional healer	Disagree	28	(4.6)	7	(2.1)	21	(7.8)	<0.01
If I got Ebola symptoms, I would hide away in my house	Disagree	57	(9.4)	8	(2.4)	49	(18.3)	<0.01
If a friend or family member gets Ebola, I would take them to a treatment center	Agree	447	(74.0)	241	(71.5)	206	(77.2)	0.12
If a friend or family member gets Ebola, I would take them to a traditional healer	Disagree	26	(4.3)	6	(1.8)	20	(7.5)	<0.01
If a friend or family member gets Ebola, I would keep them in my house	Disagree	29	(4.8)	6	(1.8)	23	(8.5)	<0.01

**Abbreviation:** MOHSW = Ministry of Health, Liberia.

* High-incidence counties = Bong and Margibi.

† Low-incidence counties = Maryland, River Gee, and Sinoe.

**TABLE 3:** Non-scored section and summary results, Knowledge, Attitudes and Practices survey — Liberia, September 17–October 11, 2014

Section/Question	Agreed among all respondents (N = 609)		Agreed in high-incidence counties* (n = 338)		Agreed in low-incidence counties† (n = 271)		p-value
		
	No.	(%)	No	(%)	No.	(%)	
**Fear of individuals**							
I am afraid of people with Ebola	582	(96.5)	328	(98.2)	254	(94.2)	0.01
I am afraid of people who live with Ebola patients	584	(96.1)	331	(97.9)	253	(93.7)	0.01
**Fear of Ebola treatment centers**							
I am afraid of treatment centers	342	(56.6)	184	(54.6)	158	(59.2)	0.26
If I got Ebola symptoms, I would be afraid of going to a treatment center	154	(25.5)	59	(17.6)	95	(35.6)	<0.01
If I go to a treatment center, I will die	110	(18.1)	37	(11.0)	73	(26.9)	<0.01

* High-incidence counties = Bong and Margibi.

† Low-incidence counties = Maryland, River Gee, and Sinoe.

## Footnotes

* Variations in numbers are related to factors such as community acceptance, weather conditions, and road access (the survey was conducted during the rainy season).
